# Comparing the Symptomatology of Post-stroke Depression with Depression in the General Population: A Systematic Review

**DOI:** 10.1007/s11065-023-09611-5

**Published:** 2023-09-05

**Authors:** J. J. Blake, F. Gracey, S. Whitmore, N. M. Broomfield

**Affiliations:** Department of Clinical Psychology and Psychological Therapies, Norwich Medical School, University of East Anglia, Norwich Research Park, Norwich, NR4 7TJ UK

**Keywords:** Post-stroke depression, Phenomenology, Symptomatology, Stroke, Systematic review

## Abstract

Previous research into the phenomenological differences of post-stroke depression (PSD) has typically focused on comparisons of symptom profiles between stroke and non-stroke population controls. This systematic review aimed to synthesize these findings with results from other methodological approaches that contribute to an understanding of phenomenological differences. Articles were identified via a systematic search of seven databases and additional manual searching. A narrative synthesis approach was adopted because of the high methodological heterogeneity. Twelve articles comparing the symptomatology of depression between stroke and non-stroke controls were included. Three distinct methodological approaches, relevant to the aim, were identified: comparisons of profiles among groups with similar overall depression severity, comparisons of the strengths of correlations between a symptom and depression, and comparisons of latent symptom severity. The symptomatology of depression was generally similar between the groups, including somatic symptoms, despite the hypothesized interference of comorbid physical stroke effects. Despite high heterogeneity, there was a tentative indication that post-stroke depression manifests with comparatively less severe/prevalent anhedonia. Possible mechanisms for the observed similarities and differences are explored, including suggestions for future research.

**Supplementary Information:**

The online version contains supplementary material available at 10.1007/s11065-023-09611-5.

## Introduction

Depression is a common consequence of stroke, occurring in one-third of survivors (Hackett & Pickles, 2014; Mitchell et al., 2017). Post-stroke depression (PSD) is associated with poorer functional outcomes, reduced social engagement, and higher rates of mortality. Accordingly, PSD must be assessed accurately so that effective and targeted interventions are made available (Deng et al., 2017; Robinson & Jorge, 2016; Towfighi et al., 2017).

Accurate assessment and support for PSD require a clear and grounded conceptualization of how it manifests in stroke survivors. However, attempts to understand the phenomenology and etiology of PSD are complicated by the wide range of morbidities after strokes, including physical and cognitive disability, functional impairment, fatigue, personality changes, and neurovascular alterations (Duncan, 1994; Hu et al., 2017; Teasdale & Engberg, 2010). Broomfield et al. (2011) outline four examples of how these factors can interact to cause and maintain depressive symptoms: (1) the impact of physical impairment on activity engagement and social participation, (2) the “depressogenic” effect of medical comorbidities and neurobiological alterations, (3) the presence of stroke-specific negative attributions, and (4) the impact of cognitive dysfunction in biasing information processing in favor of depression-reinforcing appraisals. PSD must, therefore, be understood as complex and multi-faceted, with unique interactions at the biological, psychological, and social levels (Dowswell et al., 2000; Mitchell et al., 2017; Newberg et al., 2009; Shi et al., 2017).

Concerns also exist about whether certain stroke sequelae, such as post-stroke fatigue, could be mistaken for somatic symptoms of depression, such as tiredness and feeling slowed down, or vice versa (Acciarresi et al., 2014). Furthermore, processes of post-stroke adjustment, often involving high emotional arousal, in addition to the phenomenon of post-stroke emotionalism, obfuscate the attribution of expressions of negative emotion to depression (Fitzgerald et al., 2021; Taylor et al., 2011). Comparisons of symptom profiles between depressed and non-depressed stroke groups indicate that somatic and affect-related items capture substantial variance attributable to depression, suggesting that these are symptoms of PSD, despite the overlap with other phenomena (de Man-van Ginkel et al., 2015).

Thus, it remains somewhat unclear whether PSD differs significantly from depression in non-stroke populations. For example, proponents of the vascular depression hypothesis argue that depression etiology in neurovascular disease populations may be distinct from major depression in the general population (Aizenstein et al., 2016; Alexopoulos et al., 1997), stemming from findings that neurovascular changes are independent predictors of depressive experiences (Pan et al., 2012; Thomas et al., 2004) and are associated with poorer response to treatment (Aizenstein et al., 2014). Furthermore, qualitative studies of depression in stroke populations outline experiences and narratives that appear unique to this group; for example, when stroke survivors are asked to reflect on life before and after stroke, such studies highlight themes of identity loss, loneliness in post-stroke experience, self-blame, guilt, and burden-related beliefs (Crowe et al., 2016; Taule & Råheim, 2014).

However, arguments for PSD as a distinct entity are also open to criticism. While qualitative studies reveal narratives and meanings that may be unique to stroke recovery (Crowe et al., 2016; Taule & Råheim, 2014), such work cannot indicate population-level differences. Differences in narrative or cognitive accounts of guilt between stroke and non-stroke depression might not be indicative of differences in the frequency, severity, and functional impact of guilt-related cognitions more broadly. Indeed, several studies have found evidence of similarities in depression profiles (de Man-van Ginkel et al., 2015; Gainotti et al., 1999; Lipsey et al., 1986). Studies that compare symptom profiles in this way are, however, only one of the many possible methodological approaches to the comparison of symptomatology between depressed and non-depressed groups.

Though several narrative and systematic reviews have summarized PSD risk factors, symptom correlates, and epidemiology (Backhouse et al., 2018; Gordon & Hibbard, 1997; Medeiros et al., 2020; Robinson & Jorge, 2016), no review has so far investigated the comparative phenomenology of PSD and depression in the general population using systematic search, quality rating, and data synthesis (Espárrago-Llorca et al., 2015). Comparison with non-stroke groups, as a benchmark, is essential for improving our understanding of phenomenological differences in PSD, which are otherwise challenging to contextualize when studying stroke populations alone. The aim of the current systematic review, therefore, is to answer the following research question: are there population-level differences in symptomatology between PSD and depression in the general population?

## Methods

The search was conducted in September 2021, followed by an update search in January 2022. The review was registered to Prospero on 18 August 2021 (ID: CRD42021272862). At the time of registration and submission for publication, no similar reviews were registered on Prospero or the Cochrane database. A scoping search indicated significant heterogeneity in methodology because of variation in the stroke measures used, the time elapsed since the index stroke event, methods of comparison, nationality, residential setting, and other factors. Accordingly, a narrative synthesis approach was adopted, following guidance provided by Popay et al. (2006).

### Eligibility Criteria

Eligibility criteria used the PICOS (Population, Intervention, Comparison group, Outcomes, and Study) framework and are outlined in Table 1 (Methley et al., 2014; Pollock & Berge, 2018). This review did not focus on clinical intervention, so this criterion was removed.

**Table 1:** Eligibility criteria for the inclusion of studies

	**Inclusion criteria**	**Exclusion criteria**
Population	Adults with a current diagnosis or history of stroke or strokes, of both ischemic and hemorrhagic origins	People with transient ischemic attacks (TIAs) with or without the presence of stroke. Populations with separate or additional acquired or progressive neurological conditions, such as hemorrhages secondary to traumatic brain injury, small vessel disease, or vascular dementia. Articles not written in English
Study outcome	Validated self-report quantitative measures or clinical interviews. Decisions about sufficient measure validity were based on the quality of initial validation studies and whether the measure has specific stroke or acquired brain injury validity evidence	Studies that did not contain quantitative data on depressive symptoms. Clinician-rated measures, such as the Stroke Aphasic Depression Questionnaire (SAD-Q; Sutcliffe & Lincoln, 1998), because of specific interest in this study of direct indicators of the internal world. Social factors, such as social isolation, were not of interest to the current study, having been adequately investigated by previous reviews and meta-analyses of predictors of PSD (e.g., Hackett & Anderson, 2005)
Comparison group	A comparison group of healthy individuals without neurological impairment	Comparison groups with specific health conditions, such as heart disease or orthopedic injury
Design and analysis	Any quantitative analyses that could provide valid insight into phenomenological between-group differences	Studies that statistically compared overall depression scores or compared depressive symptoms without accounting for differences in overall depression severity; secondary sources, such as book chapters, systematic reviews, or meta-analyses; studies with unextractable data

### Search Strategy

The search was completed on EBSCOhost, using the following databases: Academic Search Complete, AMED (The Allied and Complementary Medicine Database), APA PsycArticles, APA PsycInfo, CINAHL Complete (Cumulative Index of Nursing and Allied Health Literature), MEDLINE Complete, and OpenDissertations with the following keywords and MESH terms:

(“stroke” or “cerebrovascular accident*” or “post-stroke” or “subarachnoid hemorrhage” or “cerebral infarct*” or “lacunar infarct*” or “lacunar stroke” or “cerebral hemorrhage” or “Hypoxia-ischemia, Brain” or “brain infarction”) AND (“low mood” or “depress*” or “mood” or “wellbeing” or “distress*” or “affect” or “psychological distress” or “Stress, psychological” or “psychological distress” or “mental depression”) AND (“phq-9” or “phq-2” or “phq9” or “patient health questionnaire-9” or “patient health questionnaire” or “patient health questionnaire-2” or “Geriatric Depression Scale” or “GDS” or “GDS-15” or “hospital anxiety and depression scale” or “HADS” or “Center for Epidemiologic Studies Depression Scale” or “CES-D” or “Beck Depression Inventory” or “Beck Depression Inventory-II” or “BDI-II” or “BDI” or “Structured Clinical Interview for DSM-IV” or “SCID” or “SCID-II” or “The Structured Clinical Interview for DSM-5” or “Composite International Diagnostic Interview” or “CIDI” or “Diagnostic Interview Schedule” or “Mini-International Neuropsychiatric Interview” or “MINI” or “M.I.N.I” or “Aphasia Depression Rating Scale” or “ADRS” or “Brief Assessment Schedule Depression Cards” or “BASDEC” or “Montgomery–Asberg Depression Rating Scale” or “MADRS” or “Psychiatric Assessment System” or “Schedule for Affective Disorders and Schizophrenia” or “SADS” or “Schedules for Clinical Assessment in Neuropsychiatry” or “Signs of Depression Scale” or “SODS” or “Visual Analogue Mood Scale” or “VAMS” or “Hamilton Depression Rating Scale” or “HAM-D”)

A manual search was also completed by screening reference lists of the included articles, reviews, or book chapters that were relevant to the review question (e.g. Robinson, 2006).

### Screening and Selection

Articles were sequentially screened by title, abstract, and full text. A second reviewer screened 10% at each stage, blind to the ratings of the primary reviewer; a higher percentage was not possible because of resource limitations. At each stage, the primary and second reviewers discussed and resolved incidences of conflict. In cases where the primary reviewer had not considered relevant constructs or methods, the primary reviewer re-screened the excluded articles under the refined criteria.

### Quality Rating

Quality assessment was employed to assess risk of bias using the National Heart, Lung, and Blood Institute (NHLBI) Quality Assessment Tool for the Quality Assessment of Observational Cohort and Cross-sectional Studies (National Heart Lung and Blood Institute, 2013).

The NHLBI tool comprises fourteen items, with nominal responses of “Yes,” “No,” or “Other (cannot determine, not reported, not applicable).” Overall quality rating of “Good,” “Fair,” or “Poor” is based on reviewer judgment, rather than by computation. This supports flexibility in weighting items that are important for the specific methodology of the study. Papers shortlisted after full-text screening were rated for risk of bias.

### Data Extraction and Analysis

Data on participant and sample characteristics, study design, stroke characteristics (e.g., time since index stroke event and type of stroke), outcome measures used, method of analysis, and key findings were extracted from studies meeting all criteria. For each study, findings were coded in the direction of significance: studies that found greater prevalence, severity, or correlation of a symptom with depression in the stroke group compared to the non-stroke control group were coded as “more”; studies that found the reverse were coded as “less”; and non-significant findings were coded as “no difference.” If significance testing was not conducted in the source papers, recommendations of significance criteria from other papers were applied by the authors of the current paper to determine the direction of effect. For example, de Man-van Ginkel et al. (2015) did not statistically test for differences in the prevalence of symptoms and instead specified a 10% greater prevalence of a symptom as their criterion for significance in comparisons of symptom profiles. This criterion was applied to determine significance in similar papers without any reported significance test.

Categorization of the time since the index stroke event of the stroke group(s) was made for each comparison, “ < 6 weeks,” 6–12 weeks,” “12 weeks to 1 year,” and “ > 1 year,” based on approximate thresholds for recovery stages reported in the literature. Most stroke recovery is observed before 12 weeks and approaches a flattening of trajectory beyond 1 year (Douiri et al., 2017; Kwakkel, 2004).

Many symptoms were extracted because of the variability of measures used and different symptoms assessed by each measure (Cumming et al., 2010; de Man-van Ginkel et al., 2015; House et al., 1991). Dimension reduction was, therefore, performed on the extracted symptoms to consolidate them into a manageable set of broader symptom domains and to support comparisons of similar or overlapping symptoms between measures. In cases where findings for multiple symptoms loaded onto the same domain, a scoring method was used to determine the overall significance category of that new higher-order domain; all symptoms within the domain were scored + 1 for a “more” finding, −1 for “less,” and 0 for “no difference.” The summed score was divided by the number of symptoms in that category with reported findings. Combined scores between −0.5 and +0.5 were assigned “no difference,” and scores greater than ± 0.5 were assigned the category “more” or “less.” This ensured that the presence of only one “more” or one “less” finding amidst multiple “no difference” findings did not overstate the level of overall difference within that domain. This approach is consistent with the methods outlined by Thomson and Thomas (2013).

## Results

### Study Inclusion

**Fig. 1:**
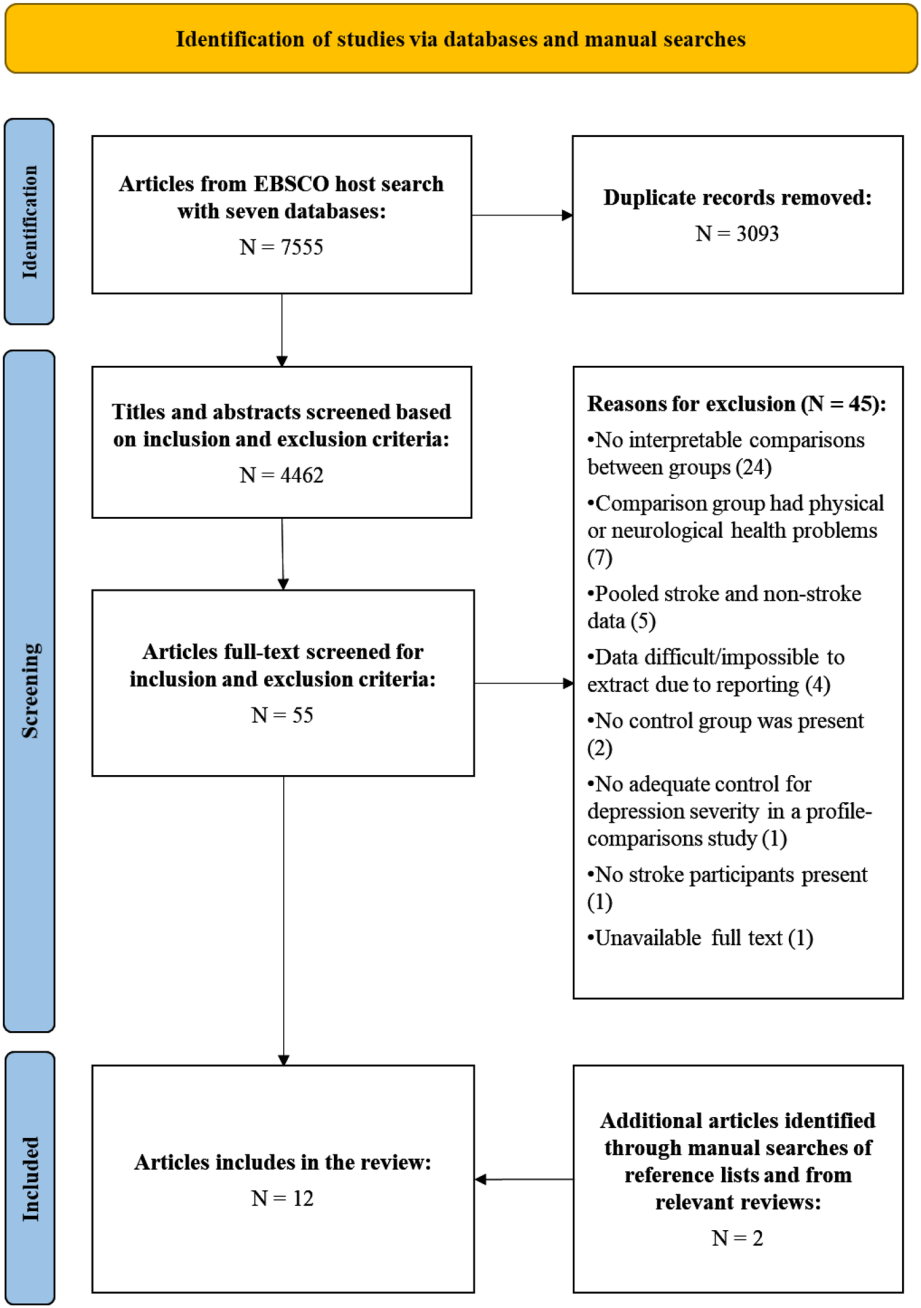
A flowchart of the article identification, screening, and selection process, adapted from the guidelines and templates published by Page et al. (2021)

From 4462 original articles identified, 58 articles were selected for full-text screening. Most articles were ineligible due to the non-reporting of statistics that allowed a valid comparison of depressive symptomology between groups. Twelve eligible studies were included for review (Fig. 1).

### Quality Assessment and Risk of Bias

Two studies were rated as “good” in quality and nine studies as “fair” (Supplementary Table 1). Two studies were allocated a “fair to poor” rating, primarily because they featured a depression measure with only limited validation evidence, the Post-Stroke Depression Rating Scale (PDRS; Gainotti et al., 1997, 1999). Despite these weaknesses, these studies were included in the analysis because the methodology was otherwise of high relevance to the research question.

### Study Details

Characteristics of each of the included studies are provided in Table 2.

**Table 2:** Study characteristics of the included articles

**Authors (year)**	**Methodological category and design**	**Quality rating**	**Participant characteristics**	**Setting**	**How was depression diagnosed and overall depression scores matched?**	**Outcome measures**	**How was significance decided?**	**Main findings (reported as questionnaire items/depression symptoms before dimension reduction)**
Gainotti et al. (1999)	**Category:** profile comparisons (symptom severity) **Design:** cross-sectional, between groups	Fair to poor	**Stroke group:** three groups with single first-time unihemispheric stroke, based on time since stroke: < 2 months (*N* = 58), 2–4 months (*N* = 52), > 4 months (*N* = 43). Only depressed people were included **Control group:** 30 mental health inpatients for endogenous depression. Demographic comparisons between the control and stroke groups were not reported. Only depressed people were included	**Stroke group:** Italian citizens. Unclear location of residence **Control group:** Italian inpatients	Depressed people were classified by researchers following a clinical interview, using the DSM-III-R and questionnaire results No statistical test for differences in overall depression scores as indicators of similar severity	Post-Stroke Depression Rating Scale (PDRS)	No significance test was used in the source paper. The source paper authors used > 1 PDRS score point difference in a symptom between groups as the criterion for difference	Findings depend on each time point since the index stroke. Data were entered separately during synthesis **Symptoms consistently (≥ 2/3 of the stroke groups) more severe in stroke:** catastrophic reactions, hyper-emotionalism, diurnal variations **Symptoms consistently (≥ 2/3 of the stroke groups) more severe in controls:** mood, suicidal ideation, anhedonia **Symptoms with no consistent difference:** vegetative disorders, apathy, anxiety, guilt
Gainotti et al. (1997)	**Category:** profile comparisons (symptom severity) **Design:** cross-sectional, between groups	Fair to poor	**Stroke group:** three groups with single first-time unihemispheric stroke (*N* = 124). The time elapsed since the onset of stroke was between 2 weeks and 6 months. Stroke patients were sorted into post-stroke major depression, minor depression, and no depression **Control group:** 17 mental health inpatients for endogenous depression, matched to the stroke group by age and education. Only depressed people were included	**Stroke group:** Italian residents. Unclear location of residence **Control group:** Italian psychiatric inpatients	Depressed people were classified by researchers following a semi-structured clinical interview, using the DSM-III-R and questionnaire results No test for differences in overall depression scores	Post-Stroke Depression Rating Scale (PDRS)	Duncan test for symptom-level comparisons in the source paper Only comparisons between post-stroke major depression and the control group were extracted, not the “post-stroke minor depression” or “no depression” samples, as only major depression had similar overall depression severity	**Symptoms more severe in stroke:** anxiety, catastrophic reactions, hyper-emotionalism, diurnal variations **Symptoms more severe in controls:** mood, suicidal ideation, anhedonia **Symptoms with no difference:** vegetative disorders, apathy
House et al. (1991)	**Category:** profile comparisons (symptom prevalence) **Design:** longitudinal, between groups	Good	**Stroke group:** 128 first-stroke patients assessed at three time points post-stroke: 1 month (*n* = 78), 6 months (*n* = 107), and 12 months (*n* = 88). Additional participants were recruited between 1 and 6 months. Forty-five percent had less than the maximum Barthel ADLs immediately post-stroke. Severity was difficult to determine. Mixed depressed and non-depressed **Control group:** 111 participants randomly sampled from general practice, using stratified random sampling approximately matched for age and sex. Mixed depressed and non-depressed	**Stroke group:** English people living in the community **Control group:** English people living in the community	Depressed people were classified via a clinical interview and Present State Examination, but the sample did not exclusively consist of those with depression Total depression scores on the Beck Depression Inventory (BDI) were not significantly different between stroke and controls at any time point post-stroke	Beck Depression Inventory (BDI); positive ratings defined a symptom as present	No significance test or other criteria for difference were used in the source paper; a prevalence ≥ 10% was used by the authors of the current paper as a criterion for significance, based on the methodology of de Man-van Ginkel et al. (2015)	Findings depend on each time point since the index stroke. Data were entered separately during synthesis **Symptoms consistently (≥ 2/3 of the stroke groups) more prevalent in stroke:** work inhibition/inability to work **Symptoms consistently (≥ 2/3 of the stroke groups) more prevalent in controls:** sleep problems, loss of libido **Symptoms with no consistent difference:** guilt, depressed mood, loss of interest, tiredness, suicidal ideation, sense of failure, self-hate, irritability, social withdrawal, indecisiveness, self-accusation, crying, body image
Lipsey et al. (1986)	**Category:** profile comparisons (symptom prevalence) **Design:** cross-sectional, between groups	Fair	**Stroke group:** 43 ischemic or hemorrhagic stroke patients were assessed less than 6 months following their stroke. All stroke patients were depressed accordingly to DSM-III criteria for major depression **Control group:** 43 “functionally depressed” participants. Control participants did not differ in any key demographic. Significantly fewer post-stroke patients reported previous psychiatric disorder	**Stroke group:** US citizens in the community or inpatient wards **Control group:** US psychiatric inpatients	Depressed patients were classified via a clinical interview using the Present State Examination and diagnosed using the DSM-III Non-significant differences in Hamilton depression total scores but significantly lower total PSE scores in stroke. Profiles had substantial visual overlap	Present State Examination (PSE)	Chi-square test of significance, used in the source paper	**Symptoms more prevalent in stroke:** slowed down **Symptoms more prevalent in controls:** loss of interest and concentration **Symptoms with no difference:** simple depression, general anxiety, affective flattening, hypomania, overactivity, special features of depression, agitation, self-neglect, ideas of reference, tension, lack of energy, worrying, irritability, social unease, other symptoms of depression
Cumming et al. (2010)	**Category:** profile comparisons (symptom prevalence) **Design:** cross-sectional, between groups	Good	**Stroke group:** 149 ischemic or hemorrhagic stroke patients assessed 18 months post-stroke, diagnosed with post-stroke depression **Control group:** 745 age- and sex-matched general population controls. Recruited from previous studies. Because they were general population participants, a small percentage (9%) had a history of stroke. All controls had a diagnosis of a major depressive episode	**Stroke group:** Swedish citizens in the community **Control group:** Swedish citizens in the community	Patients were classified as depressed according to the DSM-III-R following a clinical interview Only those with a diagnosis of depression following psychiatric interviews were analyzed. No analysis of overall depression scores among those with a diagnosis	Montgomery-Asberg Depression Rating Scale (MADRS)	Mann–Whitney *U* significance test, used in the source paper	**Symptoms more prevalent in stroke:** none **Symptoms more prevalent in controls:** inability to feel, disturbed sleep **Symptoms with no difference:** sadness, suicidal thoughts, observed sadness, inner tension, disturbed appetite, concentration difficulties, loss of energy, pessimistic thoughts
de Man-van Ginkel et al. (2015)	**Category:** profile comparisons (symptom prevalence) **Design:** cross-sectional, between groups	Fair	**Stroke group:** 54 depressed ischemic or hemorrhagic stroke patients assessed 6 to 8 weeks post-stroke, selected from a sample of 382 stroke patients. The median Barthel score was 97.5, indicating mild stroke severity. Stroke patients were categorized by depression status **Control group:** 150 depressed general practice patients, selected from a wider pool of 1160 general practice patients. Uncontrolled demographic differences were reported. Controls were categorized by depression status	**Stroke group:** Dutch citizens in the community **Control group:** Dutch citizens in the community	Patients were diagnosed as depressed using the CIDI structured interview for DSM-IV-TR classifications Significantly higher median PHQ-9 total score in stroke among depressed groups. Profiles overlapped significantly for percentage prevalence with “at least several days” in the past 2 weeks	Patient Health Questionnaire-9 (PHQ-9)	No significance test was used in the source paper; the source paper authors used a prevalence of ≥ 10% as an indicator of significance	Data were reported for non-depressed groups too, but data were only extracted for depressed groups **Symptoms more prevalent in stroke:** concentration, motor retardation, suicidal ideation **Symptoms more prevalent in controls:** anhedonia, appetite disruption, guilt **Symptoms with no difference:** depressed mood, sleep problems, tiredness/low energy
Bennett et al. (2006)	**Category:** comparative correlation strengths **Design:** cross-sectional, between groups	Fair	**Stroke group:** 79 stroke inpatients assessed 2 to 4 weeks post-stroke **Control group:** 49 healthy older adults, recruited via convenience sampling	**Stroke group:** UK subacute stroke inpatients **Control group:** UK older adults in the community	Depression diagnosis and matched depression scores are not required for correlation strength studies	**Depression measure:** Hospital Anxiety and Depression Scale-Depression Subscale (HADS-D) **Associated symptom and measure:** Self-esteem using the Visual Analog Self-esteem Scale (VASES)	Correlation coefficient (Spearman rho), used in the source paper A Fisher *r* to *z* transformation was performed by the current reviewers to test differences in correlation strengths	Correlation coefficients between depression (HADS-D) and symptom measure (VASES). Fisher *r* to *z* supports a significance test between two correlation strengths **Stroke correlation:** *r* = −0.52, *p* < 0.001 **Control correlation:** *r* = −0.54, *p* < 0.001 **Fisher *r* to *z*:** non-significant, *z* = −0.15 *p* = 0.88
Fleming et al. (2021)	**Category:** comparative correlation strengths **Design:** cross-sectional, between groups	Fair	**Stroke group:** 69 stroke participants, recruited via convenience sampling. Participants were > 3 months post-stroke **Control group:** 63 healthy older adults, recruited via convenience sampling. Controls were similar in age and sex	**Stroke group:** UK residents in the community **Control group:** UK residents in the community	N/A	**Depression measure:** Hospital Anxiety and Depression Scale-Depression Subscale (HADS-D) **Associated symptom and measure: s**leep quality/insomnia using the Sleep Condition Indicator (SCI)	A regression of SCI to HADS-D scores was reported in the source paper. If stroke status contributed to the model *r* squared, this would be interpreted as an indicator of different strength of association	The non-stroke control group independent variable did not contribute significantly to the model, indicating a non-specific difference between groups in the relationship between insomnia and depression. The authors concluded that the relationship between insomnia and depression was not specific to stroke
Schramke et al. (1998)	**Category:** comparative correlation strengths **Design:** cross-sectional, between groups	Fair	**Stroke group:** 22 participants with a single right hemisphere (RH) stroke and 22 participants with a single left hemisphere stroke (LH), recruited via convenience sampling. The mean time since stroke for the RH group was 3.6 (SD: 3.41) and LH was 3.5 (SD: 2.98) **Control group:** 24 controls, recruited via friends and family of stroke participants. Controls and stroke groups were similar in age, sex, and education level	**Stroke group:** US residents in the community **Control group:** US residents in the community	N/A	**Depression measures:** Center for Epidemiologic Studies-Depression Scale (CES-D) and the Hamilton Depression Rating Scale (HDRS) **Associated symptom and measure:** anxiety using the Beck Anxiety Inventory (BAI)	Correlation coefficient with Fisher *r* to *z* transformation, reported by the source paper	**RH stroke correlation of the BAI with CES-D:** *r*^2^ = 0.57, *p* = 0.13 **LH stroke correlation of the BAI with CES-D:** *r*^2^ = 0.72, *p* < 0.01 **Control correlation of the BAI with CES-D:** *r*^2^ = 0.85, *p* < 0.01 ***r* to *z* comparison RH to control for the BAI and CES-D:** significant difference (less associated in stroke) ***r* to *z* comparison LH to control for the BAI and CES-D:** non-significant **RH stroke correlation of the BAI with HDRS:** *r*^2^ = 0.47, *p* = 0.05 **LH stroke correlation of the BAI with HDRS:** *r*^2^ = 0.44, *p* = 0.05 **Control correlation of the BAI with HDRS:** *r*^2^ = 0.80, *p* < 0.01 ***r* to *z* comparison RH to control for the BAI and HDRS:** significant difference (less associated in stroke) ***r* to *z* comparison LH to control for the BAI and HDRS:** significant difference (less associated in stroke)
Stokes et al. (2011)	**Category:** comparative correlation strengths **Design:** cross-sectional, between groups	Fair	**Stroke group:** 69 first-stroke participants, recruited via convenience sampling. Participants were < 3 years post-stroke. Barthel index mean score: 86 (moderate impairment) **Control group:** 63 age- and gender-matched controls. No significant demographic differences between groups in sex, age, marital status, living arrangements, or type of house	**Stroke group:** Irish residents in the community **Control group:** Irish residents in the community	N/A	**Depression measure:** Geriatric Depression Scale (GDS) **Associated symptom and measure:** fatigue using the Multidimensional Fatigue Inventory (MFI) general domain	A comparison of differences in the average effect of a one-point increase in GDS score on MFI general scores, reported by the source paper Higher relative increases in MFI scores caused by higher GDS scores are indicative of a stronger correlation	**Stroke group:** a one-point increase in GDS score corresponds with a 0.1 increase in MFI general score. This is a non-significant relationship (*p* = 0.5) **Control group:** a one-point increase in GDS score corresponds with a 0.4 increase in MFI general score, reaching significance (*p* < 0.05) **Comparison:** the difference in the effect of a one-point increase in GDS between the two groups reached statistical significance (*p* < 0.05). Fatigue is less correlated with depression in the stroke group
Vickery et al. (2008)	**Category:** comparative correlation strengths **Design:** cross-sectional, between groups	Fair	**Stroke group:** 80 stroke inpatients, recruited via convenience sampling. Patients were assessed approximately 2 weeks post-stroke **Control group: 80** volunteers recruited via convenience sampling, matched for age and education. No significant demographic differences between groups in age education, sex, or race	**Stroke group:** US inpatients **Control group:** US citizens in the community	N/A	**Depression measure:** Geriatric Depression Scale (GDS) **Associated symptom and measure:** self-esteem, using the Visual Analog Self-Esteem Scale (VASES) and the Rosenberg Self-Esteem Scale (RSES)	Correlation coefficient with Fisher *r* to *z* transformation, reported by the source paper	**Stroke RSES/GDS correlation:** *r* = −0.75 **Control RSES/GDS correlation:** *r* = −0.51 ***r* to *z* comparison RSES/GDS:** significant difference (more strongly associated in stroke) **Stroke VASES/GDS correlation:** *r* = −0.77 **Control VASES/GDS correlation:** *r* = −0.65 ***r* to *z* comparison RSES/GDS:** no significant difference *p* = 0.064
Pickard et al. (2006)	**Category:** item response theory (IRT) differential item functioning (DIF) analysis **Design:** cross-sectional, between groups	Fair	**Stroke group:** 32 depressed stroke inpatients, recruited from secondary sources. Patients were assessed approximately 3 months post-stroke. The Health Utilities Index Mark 3 mean score was 0.45, indicating “severe disability” (Feng et al., 2009) **Control group:** 366 primary care depressed adults living in the community. Demographic differences were not statistically tested, but the stroke group was 27 years older and 8% higher proportion of males	**Stroke group:** Canadian inpatients **Control group:** US residents living in the community	Between-group differences in depression severity are unimportant for DIF studies, as these are accounted for by the model	**Depression measure:** Center for Epidemiologic Studies-Depression Scale (CES-D)	DIF *t*-test statistic of latent item severity between groups, reported by the source paper. *T*-statistics relate to a significance test of logit differences between groups. *p* values were not provided, but items with *p* < 0.05 are highlighted Items with an infit mean squares (MNSQ) > 1.4 were deemed to have a poor fit to the Rasch model, as specified by the source paper authors	Broadly similar hierarchies in latent symptom severity of the items as indicators of depression (*r* = 0.75). Significant item misfit was found in the stroke group for the “unfriendly,” “crying,” and “restless” items **Higher latent symptom severity in stroke:** “I felt disliked by others” (logit diff = 0.77), restlessness (logit diff = 0.61) **Higher latent symptom severity in primary care:** crying (logit diff = 0.48), appetite disruption (logit diff = 0.65) **Items with no significant difference:** unfriendly, failure, fearful, blues, effort, talked less, sad, as good as, concentration, depressed, get going, bothered, hopeful, happy, lonely, enjoy life

#### Methodology

Three distinct methodologies for indicating symptomatologic differences in depressed mood between stroke and non-stroke participants were identified: (1) comparisons of depression symptom profiles, where depression severity is approximately similar or statistically controlled between groups, (2) comparisons of correlation strengths between a depression symptom and general depression, and (3) differential item functioning (DIF) analysis using item response theory (IRT). Profile comparison studies investigated either between-group differences in percentage prevalence of positive endorsement of a depression symptom, or differences in mean symptom score (symptom severity). The profiles needed to overlap in general severity for profile comparisons to provide valid information on specific symptom differences and thus be included. This criterion was satisfied if overall/total depression scores were not statistically different between groups. If no statistical comparison was conducted, or if a significant difference was found, profile comparisons were nonetheless included if visual inspection of plotted profiles by the reviewers suggested substantial overlap and if no greater than two-thirds of symptom differences within a profile were significantly different in the same direction. For example, if greater than two-thirds of the compared symptoms were significantly more severe in the stroke group, this supermajority would suggest that the stroke group is more likely to have greater than average depression severity and therefore be ineligible. Comparative correlation studies investigated the correlation between a symptom measure, such as a self-esteem questionnaire, and scores on a depression measure, indicating the comparative importance of that symptom in explaining depression variance (Vickery et al., 2008). IRT DIF studies offer different insights into phenomenology compared to profile comparison studies or studies of differences in correlation strength; they compare differences, between groups, in the underlying severity of depression to which the item/symptom is most sensitive.

Most studies were cross-sectional, except for House et al. (1991), who explored longitudinal changes in depression profiles. Because several studies examined multiple groups (Gainotti et al., 1999; House et al., 1991; Schramke et al., 1998) and, therefore, contributed multiple comparisons, there were twenty between-group comparisons extracted from twelve studies; Vickery et al. (2008) contributed two findings, Gainotti et al. (1999) three findings, House et al. (1991) three, and Schramke et al. (1998) four. All remaining studies (Bennett et al., 2006; de Man-van Ginkel et al., 2015; Fleming et al., 2021; Gainotti et al., 1997; Lipsey et al., 1986; Stokes et al., 2011) contributed one finding each.

#### Participants

The combined studies featured 1024 stroke group and 1741 comparison group participants, with a total sample of 2765. Participants were sampled from seven countries, all western developed nations. Ethnicity was inconsistently reported and, therefore, could not be analyzed.

The time elapsed since the index stroke event varied considerably between studies, from 2 weeks to many years. Stroke participants were sampled from inpatient settings, which were generally associated with earlier recovery time points, and the community. Most studies did not investigate lateralization, except for Schramke et al. (1998). Five studies included only participants who experienced a first stroke. Stroke severity was rarely reported; three studies reported scores on the Barthel Index for Activities of Daily Living (de Man-van Ginkel et al., 2015; House et al., 1991; Stokes et al., 2011), but none used a specific indicator of stroke severity, such as the Stroke Impact Scale (Duncan et al., 1999) or National Institutes of Health Stroke Scale (Goldstein et al., 1989). Stroke sample sizes ranged from 22 (Schramke et al., 1998) to 149 (Cumming et al., 2010); sample size justification was infrequently reported (Pickard et al., 2006).

Study comparison groups were mostly community-based (9/12). The remaining three papers, all profile comparison studies, sampled depressed psychiatric inpatients (Gainotti et al., 1997, 1999; Lipsey et al., 1986). Substantial between-group demographic differences were reported in two studies: de Man-van Ginkel et al. (2015) reported significant differences in several demographic categories, including age, sex, and education level, and Pickard et al. (2006) reported substantial differences in age, sex, and nationality of the included participants. Demographic comparisons were not reported in two studies (Bennett et al., 2006; Gainotti et al., 1999). Control group sample sizes ranged from 24 (Schramke et al., 1998) to 745 (Cumming et al., 2010).

#### Measures and Symptoms

Symptom-level data were extracted from five depression measures, PHQ-9, MADRS, BDI, PSDS, and PSE, resulting in 38 symptoms. The following symptoms were combined before dimension reduction because of overlaps in questionnaire wording: (1) depressed mood and feeling down and (2) fatigue, tiredness, and low energy. Clustering decisions were made by the judgment of the reviewers and informed by evidence and theory (see Table 3).

**Table 3:** A summary of the dimensions analyzed and the constituent symptoms

**Dimension**	**Composite symptoms and measures**	**Rationale for clustering**
Negative affect	PHQ-9 (down/depressed) MADRS (observed and reported sadness, inner tension) BDI (sadness) PSDS (depressed mood) PSE (simple depression, agitation, irritability, tension)	Negative affect is seen as a core symptom of depression (Bell, 1994). It is formulated separately from cognitions in cognitive behavioral therapy (CBT; Fenn & Byrne, 2013) Factor analysis studies find that these symptoms cluster together (Clara et al., 2001; González-Blanch et al., 2018; Steer et al., 1999; Storch et al., 2004)
Anhedonia and apathy	PHQ-9 (loss of interest in doing things) MADRS (inability to feel) BDI (lack of satisfaction, loss of interest in others) PSDS (anhedonia, apathy/abulia/indifference) PSE (affective flattening, loss of interest, and concentration)	Emotional flatness is understood as a core symptom of depression (Bell, 1994) Clara et al. (2001) found that anhedonia loaded onto a separate factor to negative affect Anhedonia and apathy are correlated and often causally linked (Ang et al., 2017). Apathy was therefore added to this dimension
Negative cognitions	PHQ-9 (feeling bad about yourself) MADRS (pessimistic thoughts) BDI (guilt, pessimistic thoughts, sense of failure, self-hate, self-blame, punishment, body image) PSDS (guilt feelings) PSE (special features of depression, ideas of reference)	Negative cognitions are identified as a core component in the cognitive theory of depression (Beck, 1979) Negative cognitions have been found to form a latent factor in factor analytic studies of the BDI (Steer et al., 1999)
Somatic features	PHQ-9 (sleep, tiredness, appetite, slowed down) MADRS (sleep, reduced appetite, lassitude) BDI (sleep, tiredness, appetite, weight, libido, somatic preoccupation) PSDS (vegetative disorders) PSE (other symptoms of depression, slowness, energy)	Somatic features of depression are documented in common depression criteria (Bell, 1994) Somatic symptoms consistently form a latent factor across multiple depression measures and strongly covary (Boothroyd et al., 2019; Cumming et al., 2010; González-Blanch et al., 2018)
Behavioral features of depression	BDI (work inhibition) PSE (self-neglect)	Behavioral responses to emotional experiences are understood in cognitive theory to be a primary factor in the maintenance of depressive symptoms and a moderating factor of outcome (Ludman et al., 2003; Moorey, 2010). Therefore, these were grouped as a dimension
Cognitive features of depression	PHQ-9 (concentration) MADRS (concentration) BDI (indecisiveness)	Cognitive impairment is a commonly reported symptom of depression (Bell, 1994) and is associated with structural brain changes in neuroimaging studies (Marazziti et al., 2010). We note that indecisiveness can be caused by cognitive impairment or worry about making “incorrect” decisions. In this circumstance, indecisiveness was added to the cognitive feature dimension because, regardless of the etiology, it results in the same external cognitive outcome of problems with making decisions Symptoms of cognitive impairment have been found to cluster as a latent factor (Adams et al., 2004)
Emotional dysregulation	PSDS (catastrophic reactions, hyper-emotionalism, diurnal variations)	Emotion dysregulation, defined here as significant and rapid changes to emotional state, is not typically included in the criteria for depression diagnosis (Bell, 1994; UK National Collaborating Centre for Mental Health, 2010). However, the authors hypothesize that elevated emotional variation can be part of the experience of post-stroke major depression in some people (Gainotti et al., 1997)
Anxiety	BAI total score PSDS (anxiety) PSD (social unease, worrying)	Worrying, anxiety, and social phobia have been found to load onto a common factor on multiple measures (Lovibond & Lovibond, 1995; Smith et al., 2002)
Suicidal ideation	PHQ-9 (thoughts about harming yourself) MADRS (suicidal thoughts) BDI (suicidal thoughts/intent) PSDS (suicidal thoughts/intent)	Maintained as a separate factor because of the importance of understanding differences in this experience as part of informing safe clinical practice (Simon et al., 2013)

The PSE items “hypomania” and “overactivity” were excluded because these are not typically included in diagnostic criteria of unipolar depression (Bell, 1994; UK National Collaborating Centre for Mental Health, 2010) and because they exhibited low prevalence in both groups in the study that used this measure (Lipsey et al., 1986).

### Main Findings

The effect of moderating variables, such as time since stroke, and their potential link to results, are reported first. Subsequently, the findings of symptom comparisons are outlined. For moderating variable analysis, comparative correlation and profile comparison studies were combined, except when the methodology type itself was identified as a moderating factor. Comparative correlation and profile comparison results were expected to broadly correspond because a trait with a higher degree of correlation with depression might also be expected to have greater prevalence and severity in depressed samples. The single DIF study (Pickard et al., 2006) was excluded from this analysis because differences in latent symptom severity were judged conceptually distinct from the association of symptoms with depression or their prevalence. For subsequent analyses of symptom differences, each methodology was analyzed separately because, despite broad epistemological similarities, methodological differences might obscure more nuanced and detailed relationships.

#### Moderation of Study Characteristics

**Fig. 2:**
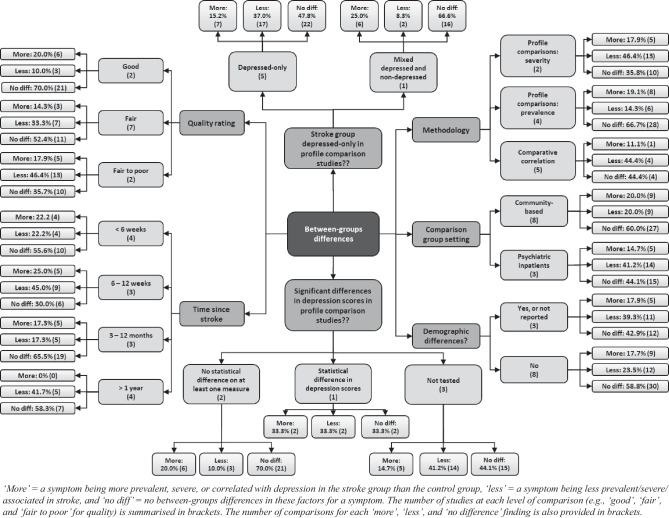
Between-group differences in “more,” “less,” and “no difference” findings, according to each moderating factor, for profile comparisons and comparative associations

Nineteen comparisons were extracted from the eleven profile comparison and comparative correlation studies. The proportions of “more” (i.e., a symptom that is more severe, prevalent, or associated with depression in the stroke group), “less,” and “no difference” findings were stratified across each level of the predictor variables of interest, for example, for each quality rating category, as summarized in Fig. 2.

##### Methodology

Comparative correlation studies reported broadly consistent results to severity-based profile comparison studies. By contrast, prevalence-based profile studies were more likely to find no differences between groups and less likely to report findings of “less” (i.e., less prevalent, severe, or associated in the stroke group).

Comparative association studies contributed far fewer results, reporting only one symptom domain per study group comparison, versus an average of seven symptom domains for profile studies. Comparative association studies also reported a narrower spectrum of symptoms, with findings only extracted for anxiety, somatic features, and negative cognitions.

##### Study Quality

Higher-rated studies were less likely to report significant differences in either direction. There was a sequential increase in the proportion of “less” findings from “high” to “fair to poor” quality, from 10 to 46.4%, respectively. Poorer quality studies, therefore, potentially underestimate symptom severity, prevalence, or association with depression in the stroke groups. All four “fair to poor” comparisons sampled psychiatric inpatients for their comparison group. Similar to the findings for overall quality, studies with uncontrolled demographic differences reported more “less” findings, 39.3% versus 23.5%, but a similar proportion of “more” findings, 17.9% versus 17.7%.

##### Time Since Stroke

The proportion of “more” findings progressively decreased with increased time since stroke. There was no discernible pattern for “less” or “no difference” findings.

##### Comparison Group Setting

Studies sampling psychiatric inpatients in their non-stroke comparison groups were twice as likely to report findings of less prevalence, severity, or association with depression in the stroke group than studies with community-based samples (41.2% versus 20%). All comparisons of suicidal ideation in non-stroke samples of inpatients were categorized as “less,” compared with 0% in the community-based samples. Studies featuring inpatient comparison groups with matched total depression severity may reflect profile differences at the more severe end of post-stroke and non-stroke depression. The residential setting of the stroke group was not analyzed because this had high correspondence with time since stroke.

##### Depressed-Only Versus Mixed Groups

Five profile comparison studies featured groups of only depressed participants, diagnosed by the researchers via clinical interview (Cumming et al., 2010; de Man-van Ginkel et al., 2015; Gainotti et al., 1997, 1999; Lipsey et al., 1986), and one included a mixture of depressed and non-depressed participants (House et al., 1991). Depressed-only samples provide greater specificity in identifying depression symptomatologic differences, rather than differences in general population characteristics. The mixed depression/non-depression study found substantially fewer “less” findings compared with those that only evaluated differences in groups with depression.

##### Differences in Indicators of Depression Severity

Three out of six profile-based studies conducted a statistical analysis of overall depression severity, through a comparison of total scores (de Man-van Ginkel et al., 2015; House et al., 1991; Lipsey et al., 1986). House et al. reported no significant differences in BDI total scores between stroke and non-stroke. Lipsey et al. reported no difference in HAM-D scores between groups but significantly higher Present State Examination scores in the non-stroke group. de Man-van Ginkel et al. reported significantly higher PHQ-9 scores in the depressed stroke group compared with the depressed non-stroke group. The remaining studies did not report total score data in the specific groups that were compared (Cumming et al., 2010; Gainotti et al., 1997, 1999). Profile plots of all six profile comparison studies were deemed by the researchers to possess substantial visual overlap, and no more than two-thirds of symptoms were significantly different in the same direction in any profile (see Supplementary Table 2).

The two studies that found no significant differences in total scores on at least one depression measure were, in general, more likely to find no difference in domain-specific comparisons between the groups (70% of comparisons). Studies that did not report overall depression scores and the study that reported a significant difference found more “less” results (41.2% and 33.3%, respectively, compared with 10% for those with no significant between-group difference in total scores). These results imply that profile studies with matched depression scores are less likely to find phenomenological differences.

#### Profile Comparison Studies

**Fig. 3:**
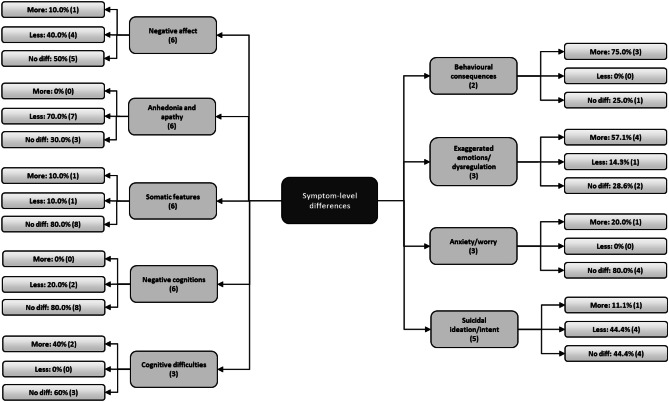
The proportion of “more,” “less,” and “no difference” findings (absolute number in brackets) for each symptom domain. The number of studies contributing data to each domain is also listed in brackets

Inferences relating to phenomenological differences were drawn by determining the percentage of “more,” “less,” and “no difference” findings for each of the nine domains (see Fig. 3 and Supplementary Table 2). Ten profile comparisons were extracted from the six profile comparison studies.

##### Negative Affect

There were mixed findings for negative affect, with 5 of 10 comparisons yielding non-significant differences. Forty percent of the comparisons found that negative affect was less severe in the stroke group, but these findings came from two papers that used psychiatric inpatients as a comparison group and compared severity, rather than prevalence (Gainotti et al., 1997, 1999).

##### Anhedonia and Apathy

Seven out of the 10 comparisons (70%) indicated less prevalence/severity of anhedonia and apathy in the stroke group, with no studies indicating greater prevalence or severity. The three “no difference” findings were extracted from one of the two papers with mixed depressed and non-depressed groups. Among those meeting criteria for depression, stroke participants experience less anhedonia and apathy.

##### Somatic Features

In 80% of cases, no difference between groups was found. When exploring individual symptom differences, “less” findings were generally due to findings of less prevalent/severe sleep disruption and lost libido in stroke. Sleep disruption was less prevalent in stroke in three of the five comparisons that featured a sleep item and no different in the remaining two. The single “more” finding was due to more prevalent appetite disruption and somatic preoccupation in the 1-month post-stroke group (House et al., 1991).

##### Negative Cognitions

The groups did not significantly differ in the presence/severity of indicators of negative cognitions. Both “less” findings were explained by a greater prevalence/severity of guilt-related cognitions in the comparison group. Pessimism was more prevalent in two of the three House et al. (1991) stroke groups, but overall negative cognition prevalence/severity was balanced out by “no difference” findings of other symptoms and a “less” finding for self-blame in one group.

##### Cognitive Difficulties

Cognitive impairment was more frequently reported in stroke in two of the five comparisons, pertaining to a higher prevalence of self-reported reduced concentration (PHQ-9) and indecisiveness (BDI) and no different in the remaining three, also relating to concentration and indecisiveness.

##### Behavioral Features

Three-quarters of behavioral symptom comparisons indicated that behavioral features of depression were more common after stroke than in comparison groups. All three “more” findings were due to the “work inhibition” item in the BDI (House et al., 1991). In the single study investigating self-neglect, no difference was observed (Lipsey et al., 1986).

##### Exaggerated Emotions/Emotional Dysregulation

Greater severity and prevalence of symptoms of emotional dysregulation were found in the stroke group in most comparisons (57.1%). All “more” findings were attributable to the PSDS measure (Gainotti et al., 1997, 1999). Surprisingly, House et al. (1991) found a lower prevalence of crying in the 1-month post-stroke group, compared to healthy controls, despite the suspected loading of post-stroke emotionalism and adjustment processes onto this item.

##### Suicidal Ideation/Intent

Suicidal ideation was less prevalent in the stroke group in 44% of cases and no different in an additional 44% of comparisons. All four “less” findings were attributable to studies that used psychiatric inpatients as comparison groups (Gainotti et al., 1997, 1999).

##### Anxiety/Worry

Anxiety was found to be more severe in stroke in one comparison using a measure assessing cognitive, somatic, and psychomotor symptoms of anxiety (Gainotti et al., 1997), but there was no evidence for a significant difference in severity or prevalence in the remaining four comparisons.

#### Profile Comparison Synthesis in Selected Studies

Differences in each symptom domain were explored within selected subgroups in the following categories:

##### Only High-Quality Studies

The two studies rated as high quality (Cumming et al., 2010; House et al., 1991) constituted four comparisons, with “no difference” in the domains of negative affect, anhedonia/apathy, somatic features, negative cognitions, cognitive dysfunction, emotion dysregulation, and suicide. Work disruption was more prevalent in all three House et al. comparisons. No symptom domains were less prevalent in stroke among high-quality studies.

##### Only Matched Overall Depression Scores

The results reported by the two papers with non-significant differences in total depression scores (House et al., 1991; Lipsey et al., 1986) were similar to the high-quality studies, with majority “no difference” findings in all domains except for behavioral disruption/work inhibition.

##### Only Including Depressed Stroke and Non-stroke Participants

All profile comparison studies featured only depressed participants, except for House et al. (1991). Among these studies, emotional dysregulation was more prevalent/severe in stroke in 100% of comparisons, and cognitive dysfunction was more prevalent in one of the two comparisons, with the other showing no difference. Anhedonia was less prevalent/severe in 100% of comparisons, negative affect in 57%, and suicidal ideation in 66.7%. Somatic features, negative cognitions, behavioral consequences, and anxiety were no different in the majority of comparisons.

When excluding the papers rated fair to low (Gainotti et al., 1997, 1999), anhedonia remained less prevalent/severe in 100% of this subset of comparisons (Cumming et al., 2010; de Man-van Ginkel et al., 2015; Lipsey et al., 1986). However, negative affect was no different in 100% of these remaining comparisons, and suicidal ideation was more prevalent in one comparison and less prevalent in the other. No other domains changed in overall majorities.

#### Comparative Correlation Strength Studies

Comparative correlation studies reported findings for insomnia (Fleming et al., 2021), fatigue (Stokes et al., 2011), self-esteem (Bennett et al., 2006; Vickery et al., 2008), and anxiety (Schramke et al., 1998). A stronger degree of correlation is interpreted to indicate that a symptom is a greater predictor of depression and therefore more central to the phenomenology of depression in that population. All comparative correlation studies were rated as fair in quality.

##### Somatic Features

The single study investigating comparative correlation strengths for insomnia found no differences in association with depression (Fleming et al., 2021). A single study found a weaker association between depression and domain-general fatigue in the stroke group (Stokes et al., 2011), by comparing differences in the effect of a one-point increase in fatigue scores on depression. The stroke group was exclusively > 1-year post-stroke.

##### Negative Cognitions (Self-Esteem)

Two studies investigated comparative correlation strengths of self-esteem with depression (Bennett et al., 2006; Vickery, et al., 2008). Vickery used two separate self-esteem measures, the RSES and the VASES, finding a greater relationship between self-esteem and depression in the stroke group using the RSES, but no difference in association using the VASES. Bennett et al. reported no difference in correlation strength, also using the VASES.

##### Anxiety

Schramke et al. (1998) contributed four comparisons for anxiety, based on two depression measures, the CES-D and the HDRS, and two stroke groups, a right-hemisphere and left-hemisphere stroke group. In three of the four comparisons, anxiety was less related to depression in the stroke groups than in the control group. There was a non-significant difference in association in the comparison featuring left hemisphere stroke patients and the CES-D as a measure.

#### Item Response Theory

The single IRT study found that feeling disliked by others and feelings of restlessness were indicative of more severe depression in the stroke group, and the presence of crying and appetite disruption was indicative of more severe depression in the primary care group (Pickard et al., 2006). No differences were found in the remaining CES-D items. Poor model fit was found for “unfriendly,” “crying,” and “restless” items in the stroke group only, suggesting that these symptoms might be less specific to experiences of depression in this group.

## Discussion

This systematic review aimed to identify similarities and differences in depression phenomenology between stroke survivors and people in the general population. Three distinct methodologies, capable of contributing to this aim, were identified by this review: comparisons of profiles among groups with similar overall depression severity, comparisons of the strengths of correlations between a symptom and depression, and comparisons of latent symptom severity using DIF. Observed moderating factors included study design/methodology, risk of bias, time since stroke, and residential setting of the control group. Notably, the two higher-quality studies were less likely to report differences in phenomenology, but one of these did not exclusively examine depressed participants. Thus, careful synthesis of patterns among subgroups of studies was required.

Across the included studies, broad similarities in the symptomology between stroke and non-stroke were found for negative affect, somatic symptoms, negative cognitions, cognitive dysfunction, and suicidal ideation. We found tentative evidence for less severity/prevalence of anhedonia, a weaker association of anxiety with depression, and a lower latent severity of crying and appetite disruption in the stroke group. A greater prevalence and/or severity of emotional dysregulation and work disruption and a greater latent symptom severity of feeling disliked and restlessness in the stroke group were also observed.

Some of the above-outlined differences were not evident when selecting only the following categories of study: those with high quality (low threat to internal validity), profile comparison studies that matched depression scores, and profile comparison studies that exclusively examined depressed participants. Among high-quality studies and those that matched depression scores, the only consistent difference was greater reported work disruption in stroke, notably with no difference in the prevalence or severity of anhedonia. However, when exclusively synthesizing profile studies that only compared depressed participants, anhedonia was less prevalent in stroke in 100% of reported comparisons, even excluding the studies classified as “fair to poor” in quality. None of these subsets compared emotion dysregulation.

The consistent absence of differences in the prevalence or severity of somatic items contradicts the common assertion that interference from physical health consequences, such as post-stroke fatigue and physical disability, undermines the reliable measurement of the somatic features of depression (Cumming et al., 2010). This finding supports previous evidence that depression contributes unique variance to these items in both groups (de Man-van Ginkel et al., 2015; Robinson, 2006) and is consistent with findings that somatic items in depression questionnaires often load onto a single latent factor in stroke (Dong et al., 2022; Katzan et al., 2021). It is possible that there is a weaker correlation between depression and somatic problems in stroke, but that this reduced correlation is offset by the presence of elevated baseline somatic symptoms, leading to a canceling out between groups. When interpreting the finding of lower sleep disruption in stroke, Cumming et al. (2010) suggest that stroke patients may experience less sleep impairment because their fatigue leads to improved sleep. Only a few studies have directly compared the prevalence of insomnia in stroke versus age-matched controls, controlling for depression severity (Fleming et al., 2021), meaning it remains difficult to validate this hypothesis. It should be noted that the prevalence of insomnia in the UK is high in the general population as well as in stroke (Baylan et al., 2020; Morphy et al., 2007). This might explain why findings appear to be inconsistent between studies on this matter (e.g., Cumming et al., 2010; Fleming et al., 2021; House et al., 1991).

The finding of lower prevalence/severity of anhedonia in stroke in all profile studies that only included depressed participants is surprising, given the evidence in support of apathy as a stroke sequela (Jorge et al., 2010). By contrast, the stroke groups presented with greater severity of problems with emotional dysregulation in 57% of comparisons, and crying was found to have lower symptom severity and poor fit in stroke, suggesting that crying is reported more readily and correlates less with depression in this group (Pickard et al., 2006). Combined, these findings provide a tentative indication that depression in the general population is more strongly associated with dulled affect and low motivation, and the post-stroke experience may be associated more strongly with emotional dysregulation. This picture is complicated by the distinct phenomena of post-stroke emotionalism (Calvert et al., 1998; Fitzgerald et al., 2021) and processes of emotional adjustment to loss (Taylor et al., 2011), in addition to the heterogeneity between studies. It is possible that the presence of elevated emotionality from adjustment and emotionalism loads negatively onto items of anhedonia because strong or changeable emotions might counteract the perception or experience of emotional flatness. Indeed, a mechanism in the opposite direction has been proposed, whereby stroke subgroups presenting with low motivation/drive present with relatively few emotional dysregulation difficulties, owing to damage to their energization system, the system responsible for the initiation and maintenance of behavior (Salas et al., 2019; Stuss, 2011). This would suggest the existence of an inverse association between these traits. Alternatively, it could be that the focus on physical recovery and return to “normal life” after stroke protects against loss of interest or reduced sense of accomplishment (Townend, 2005). Given evidence for the importance of lesion location and lateralization in presentations of both anhedonia and emotional dysregulation (Douven et al., 2017; Hackett & Pickles, 2014), greater clarity about these findings may have been possible if these stroke characteristic data were available in the reviewed studies.

The higher prevalence of cognitive complaints in the stroke groups in two out of five studies, and half of the fair- or higher-quality studies that only examined depressed participants, might be confounded by neurologically driven cognitive deficits post-stroke (Vataja et al., 2003). Studies that compare depressed and non-depressed stroke patients do, however, indicate an overlay of depression onto cognitive items (de Man-van Ginkel et al., 2015), such as impairment in concentration. However, the interaction between these two sources of impairment requires further investigation.

The trend of fewer “more” findings with elapsed time since stroke may be explained by methodological factors, such as the absence of profile comparison studies in the > 1-year post-stroke range and the observation that comparative correlation strength studies found fewer “more” results than prevalence-based studies. Stroke-related factors, such as elevated emotion during early adjustment (Taylor et al., 2011), stroke recovery (Wade et al., 1985), or recovery of post-stroke emotionalism (Morris et al., 1993), are also possible. Unexpectedly, House et al. (1991) found that crying was *less* prevalent in early recovery, contrary to theories and longitudinal data on the natural course of emotionalism (Broomfield et al., 2022; Fitzgerald et al., 2021).

This review was the first to synthesize multiple distinct methodologies to identify phenomenological differences in PSD while considering the myriad of extraneous factors that can load onto commonly used indicators of depression, such as post-stroke emotionalism (Calvert et al., 1998). A further strength was the openness of our search strategy, which enabled the identification of many relevant methodological approaches. Previous reviews have often focused on profile comparison studies (e.g., Espárrago-Llorca et al., 2015). Comparisons of correlation strengths have the advantage of identifying the degree of “closeness” of a symptom to the depression and are more robust to the confounds of profile comparisons, despite contributing less information per study. DIF offers powerful insights into differences in the relative severity of depression symptoms, a different perspective to that offered by the other methodologies.

Despite these strengths, several limitations of the review should be highlighted. First, many of the included studies were only fair or below quality, often because of a lack of control for demographic differences, non-reporting of demographics altogether, or low sample sizes. Similarly, profile studies often did not match for overall depression severity, or report overall depression severity, forcing reliance on visual inspection of profile graphs for similarity. This, in turn, could have biased the findings of similarities and differences. Though we analyzed differences in findings between quality categories to account for this bias, the picture was further obscured because one of the high-quality studies did not exclusively focus on depressed participants. For these reasons, confidence in the conclusions is limited by the quality of the studies included. Second, though judgments were agreed upon across the review team, we acknowledge that high methodological heterogeneity forced the interpretation of symptom differences and the significance of moderating factors to be based on qualitative and subjective judgments. Alternative conclusions from the same findings are thus possible. Furthermore, we acknowledge that there are conceptual issues with comparing “like for like” depression severity; for example, if there is loading from extraneous non-depression-related factors, such as post-stroke emotionalism, this might also mean that the underlying depression severity is not similar between groups, limiting comparability. Finally, due to resource limitations, quality ratings were only completed by the primary reviewer, potentially reducing the accuracy of quality assessment.

Our clinical recommendations primarily relate to the findings of greater prevalence and severity of emotional dysregulation and work disruption in stroke, as these symptom differences were consistently reported across studies, including those of higher quality. We, therefore, recommend an augmented approach to traditional therapeutic support for depression, which integrates our findings of difficulties with emotional dysregulation, impact on work, and other known relevant factors, such as psychological adjustment (Taylor et al., 2011). For emotion regulation difficulties, support with the development of intrapersonal and behavioral emotion regulation skills tailored to the patient’s neurocognitive profile, in keeping with Gross’ process model of emotion regulation in brain injury, may be a helpful augmentation to psychological therapy for depression (Gross, 1998; Salas et al., 2019). Indeed, emotion regulation difficulties in brain injury were found to be the main predictor of depression, anxiety, and distress in a study using principal component analysis (Shields et al., 2016). Broomfield et al. (2011) also recommend the consideration of additional grief work and motivational interviewing in the context of personal loss and psychological adjustment. Clinicians are, therefore, encouraged to adopt a curious approach to formulating the causes and effects of somatic and cognitive symptoms, given the evidence for person- and stroke-specific interactions of cognitive dysfunction and executive dysfunction with mood problems (Salas et al., 2013).

Regarding future research, this review has highlighted a relative scarcity of high-quality studies examining this topic, highlighting a need for further work to improve internal validity when applying these methodologies. This may include controlling for demographic differences, use of longitudinal designs, and matching depression severity, when appropriate. The presence of only one study utilizing IRT DIF comparisons to elucidate phenomenological differences indicates that this methodology is currently under-utilized (Pickard et al., 2006). Clinicians could use such insights to identify whether the presence of certain symptoms should be interpreted as more concerning than others and if this varies between populations. Further research is required to understand whether the findings of similar somatic profiles relate to the robustness of these items to extraneous sources of variance, or other mechanisms. Finally, a greater exploration of the reasons for the lower severity/prevalence of anhedonia in PSD is needed.

## Conclusions

Here, we have presented the first synthesis of phenomenological comparisons of depression between stroke and the general population. We identified three unique methodologies that can contribute to this research question. This indicates that phenomenological comparisons cannot be understood from comparisons of profiles alone and that we must consider differences in symptom prevalence, severity, “closeness” to the construct of depression, and differences in the latent severity of symptoms as indicators of depression. There were broad similarities in most domains. There was tentative evidence that, unexpectedly, anhedonia and apathy are less prevalent and/or severe in stroke compared to general population depression, although this finding was not endorsed by the majority of comparisons in high-quality studies. Anxiety was less correlated with depression in stroke. Emotional dysregulation and disruption to work were more prevalent/severe in depression after stroke and feeling disliked, and restlessness may have a higher latent depression severity in stroke. The heterogeneity of methods in the included studies limited our ability to draw definitive conclusions. A more detailed understanding of observed differences, and of mechanisms that help to integrate findings between each methodology, requires future research.

## Supplementary Information

Below is the link to the electronic supplementary material.

Supplementary file1 (DOCX 37 KB)

Supplementary file2 (DOCX 22 KB)

## Data Availability

The datasets generated during and/or analyzed during the current study are available from the corresponding author on reasonable request.
