# Supplementary material for: Comparing the Symptomatology of Post-stroke Depression with Depression in the General Population: A Systematic Review
Source: Neuropsychol Rev. 2023 Sep 5;34(3):768–90. doi: 10.1007/s11065-023-09611-5 (PMC11473539; doi:10.1007/s11065-023-09611-5)
Supplement: Supplementary file 1 — Supplementary file1 (DOCX 37 KB) [file 11065_2023_9611_MOESM1_ESM.docx]

**Supplementary Table 1** NHLBI Quality Assessment Tool ratings for included articles

|  | **Gainotti et al. (1999)** | **Gainotti et al. (1997)** | **House et al. (1991)** | **Lipsey et al. (1986)** | **Cumming et al. (2010)** | **de Man-van Ginkel et al. (2015)** | **Bennett et al. (2006)** | **Fleming et al. (2021)** | **Schramke et al. (1998)** | **Stokes et al. (2011)** | **Vickery et al. (2008)** | **Pickard et al. (2006)** |
| --- | --- | --- | --- | --- | --- | --- | --- | --- | --- | --- | --- | --- |
| 1. Was the study question or objective clearly stated? | Yes | Yes | Yes | Yes | Yes | Yes | Yes | Yes | Yes | Yes | Yes | Yes |
| 2. Was the study population clearly specified and defined? | Yes | Yes | Yes | Yes | Yes | Yes | Yes | Yes | Yes | Yes | Yes | Yes |
| 3. Was the participation rate of eligible persons at least 50%? | Yes | NR | Yes | NR | Yes | NR | NR | Yes | NR | NR | NR | NR |
| 4. Were all the subjects selected or recruited from the same or similar populations (including the same time period)? Were inclusion and exclusion crtieria for being in the study prespecified and applied uniformly to all participants? | NR | Yes | Yes | Yes | Yes | No | No | Yes | Yes | Yes | Yes | No |
| 5. Was the sample size justification, power description, or variance and effect estimates provided? | No | No | No | No | No | No | No | No | No | No | No | Yes |
| 6. For the analyses in this paper, were the exposure(s) of interest measured prior to the outcome(s) being measured? [Exposure = Stroke] | Yes | Yes | Yes | Yes | Yes | Yes | Yes | Yes | Yes | Yes | Yes | Yes |
| 7. Was the timeframe sufficient so that one could reasonably expect to see an association between exposure and outcome if it existed? | Yes | Yes | Yes | Yes | Yes | Yes | No | Yes | CD | Yes | No | Yes |
| 8. For exposures that can vary in amount or level, did the study examine different levels of the exposure as related to the outcome (e.g., categories of exposure, or exposure measured as continuous variable)? | Yes | Yes | Yes | Yes | Yes | Yes | Yes | Yes | Yes | Yes | Yes | Yes |
| 9. Were the exposure measures (independent variables) clearly defined, valid, reliable, and implemented consistently across all study participants? | Yes | Yes | Yes | Yes | Yes | Yes | Yes | Yes | Yes | Yes | Yes | Yes |
| 10. Was the exposure(s) assessed more than once over time? [replaced with outcome assessed over time] | No | Yes | Yes | No | No | No | No | No | No | No | No | No |

| 11. Were the outcome measures (dependent variables) clearly defined, valid, reliable, and implemented consistently across all study participants? | No | No | Yes | Yes | Yes | Yes | Yes | Yes | Yes | Yes | Yes | Yes |
| --- | --- | --- | --- | --- | --- | --- | --- | --- | --- | --- | --- | --- |
| 12. Were the outcome assessors blinded to the exposure status of participants? | Yes | NR | NR | NR | NR | NR | NR | NR | NR | NR | NR | Yes |
| 13. Was loss to follow-up after baseline 20% or less? | N/a | N/a | Yes | N/a | N/a | N/a | N/a | N/a | N/a | N/a | N/a | N/a |
| 14. Were key potential confounding variables measured and adjusted statistically for their impact on the relationship between exposure(s) and outcome(s)? | No | No | Yes | Yes | Yes | No | No | Yes | Yes | Yes | Yes | No |
| **Quality rating (Good, Fair, Poor)** | Fair to Poor | Fair to Poor | Good | Fair | Good | Fair | Fair | Fair | Fair | Fair | Fair | Fair |

|  | Yes |
| --- | --- |
|  | No |
|  | CD (cannot determine) |
|  | NA (not applicable) |
|  | NR (not reported) |
