# Supplementary material for: Comparing the Symptomatology of Post-stroke Depression with Depression in the General Population: A Systematic Review
Source: Neuropsychol Rev. 2023 Sep 5;34(3):768–90. doi: 10.1007/s11065-023-09611-5 (PMC11473539; doi:10.1007/s11065-023-09611-5)
Supplement: Supplementary file 2 — Supplementary file2 (DOCX 22 KB) [file 11065_2023_9611_MOESM2_ESM.docx]

| Study | Negative affect | Anhedonia and apathy | Somatic features | Negative cognitions | Cognitive | Behavioural consequences | Emotion dysregulation | Anxiety | Suicide | Percent “more” | Percent "less" | Percent "no difference" |
| --- | --- | --- | --- | --- | --- | --- | --- | --- | --- | --- | --- | --- |
| House et al. (1991) | More | No diff | No diff | No diff | More | More | No diff | NA | No diff | 37.5% | 0.0% | 62.5% |
|  | No diff | No diff | More | No diff | No diff | More | Less | NA | No diff | 25.0% | 12.5% | 62.5% |
|  | No diff | No diff | Less | No diff | No diff | More | No diff | NA | No diff | 12.5% | 12.5% | 75.0% |
| Cumming et al (2010) | No diff | Less | No diff | No diff | No diff | NA | NA | NA | No diff | 0.0% | 16.7% | 83.3% |
| de Man-van Ginkel et al. (2015) | No diff | Less | No diff | Less | More | NA | NA | NA | More | 33.3% | 33.3% | 33.3% |
| Lipsey et al (1986) | No diff | Less | No diff | No diff | NA | No diff | NA | No diff | NA | 0.0% | 16.7% | 83.3% |
| Gainotti et al (1999) | Less | Less | No diff | No diff | NA | NA | More | No diff | Less | 14.3% | 42.9% | 42.9% |
|  | Less | Less | No diff | No diff | NA | NA | More | No diff | Less | 14.3% | 42.9% | 42.9% |
|  | Less | Less | No diff | No diff | NA | NA | More | No diff | Less | 14.3% | 42.9% | 42.9% |
| Gainotti et al (1997) | Less | Less | No diff | Less | NA | NA | More | More | Less | 28.6% | 57.1% | 14.3% |
| Percent “more” | 10.00% | 0.00% | 10.00% | 0.00% | 40.00% | 75.00% | 57.14% | 20.00% | 11.11% | NA | NA | NA |
| Percent “less” | 40.00% | 70.00% | 10.00% | 20.00% | 0.00% | 0.00% | 14.29% | 0.00% | 44.44% | NA | NA | NA |
| Percent “no difference” | 50.00% | 30.00% | 80.00% | 80.00% | 60.00% | 25.00% | 28.57% | 80.00% | 44.44% | NA | NA | NA |

**Supplementary Table 2** Symptom difference findings reported by profile comparison studies

*Ten comparisons were reported, across six included profile comparison studies. Similarity in overall depression severity was judged from visual plots of profiles and also the percentage figures in the three rightmost columns. If two-thirds or more of the total comparisons were either “more” or “less”, this would indicate consistent difference in one direction and therefore differences in overall severity. None were excluded because of this criterion. The 50% threshold was only crossed in the Gainotti et al (1997) paper. The percentages reported in the bottom three rows are the percentage of findings for each symptom, also reported in Figure 2.*
